# Multi-UAV-Assisted Task Offloading and Trajectory Optimization for Edge Computing via NOMA

**DOI:** 10.3390/s25164965

**Published:** 2025-08-11

**Authors:** Jiajia Liu, Haoran Hu, Xu Bai, Guohua Li, Xudong Zhang, Haitao Zhou, Huiru Li, Jianhua Liu

**Affiliations:** 1 Faculty Development and Teaching Evaluation Center, Civil Aviation Flight University of China, Guanghan 618307, China; 2 Institute of Electronic and Electrical Engineering, Civil Aviation Flight University of China, Guanghan 618307, China; huhaoran@cafuc.edu.cn (H.H.); baixugood@cafuc.edu.cn (X.B.); liguohua@cafuc.edu.cn (G.L.); xudonggood@cafuc.edu.cn (X.Z.); zhouhaitao@cafuc.edu.cn (H.Z.); jianhuacafuc13@cafuc.edu.cn (J.L.); 3 Flight Training Center of Civil Aviation Flight University of China, Guanghan 618307, China; lihuiru@cafuc.edu.cn

**Keywords:** edge computing, UAV, NOMA, task offloading, trajectory optimization

## Abstract

Unmanned Aerial Vehicles (UAVs) exhibit significant potential in enhancing the wireless communication coverage and service quality of Mobile Edge Computing (MEC) systems due to their superior flexibility and ease of deployment. However, the rapid growth of tasks leads to transmission queuing in edge networks, while the uneven distribution of user nodes and services causes network load imbalance, resulting in increased user waiting delays. To address these issues, we propose a multi-UAV collaborative MEC network model based on Non-Orthogonal Multiple Access (NOMA). In this model, UAVs are endowed with the capability to dynamically offload tasks among one another, thereby fostering a more equitable load distribution across the UAV swarm. Furthermore, the integration of NOMA is strategically employed to alleviating the inherent queuing delays in the communication infrastructure. Considering delay and energy consumption constraints, we formulate a task offloading strategy optimization problem with the objective of minimizing the overall system delay. To solve this problem, we design a delay-optimized offloading strategy based on the Twin Delayed Deep Deterministic Policy Gradient (TD3) algorithm. By jointly optimizing task offloading decisions and UAV flight trajectories, the system delay is significantly reduced. Simulation results show that, compared to traditional approaches, the proposed algorithm achieves a delay reduction of 20.2%, 9.8%, 17.0%, 12.7%, 15.0%, and 11.6% under different scenarios, including varying task volumes, the number of IoT devices, UAV flight speed, flight time, IoT device computing capacity, and UAV computing capability. These results demonstrate the effectiveness of the proposed solution and offloading decisions in reducing the overall system delay.

## 1. Introduction

With the rapid growth of Internet of Things (IoT) devices, how to process large volumes of distributed data efficiently at the network edge has become an urgent challenge for wireless communications. Mobile Edge Computing (MEC) is widely seen as an effective way to offload computation and ease the resource constraints of IoT devices. However, fixed edge servers often struggle to adapt in dynamic environments or remote areas [1]. In this context, Unmanned Aerial Vehicles (UAVs) have gained attention for expanding MEC coverage thanks to their strong mobility, flexible deployment, wide-area coverage, and relatively low cost. Especially when ground infrastructure is lacking or out of service, UAVs can quickly set up temporary computing and communication support [1,2,3,4,5].

Although UAV-assisted MEC has been studied extensively, many existing works still rely on the assumption of a single UAV acting as a flying base station or computing node. This approach falls short when dealing with large-scale, highly dynamic IoT environments, where sudden spikes in workload or coverage gaps are common. Moreover, most prior studies tackle either communication resource allocation or flight trajectory planning in isolation, and rarely consider how to jointly coordinate UAV movement, user access, and task distribution in a unified, adaptive way. Therefore, how to design a joint task offloading and trajectory optimization strategy in a multi-UAV collaborative MEC network, which supporting high user concurrency and optimize the overall system delay is still an severe problem.

To address the obove problem, we propose a joint task offloading and trajectory control scheme. We frame this as a Markov Decision Process (MDP) and design an algorithm based on the Twin Delayed Deep Deterministic Policy Gradient (TD3). The algorithm can adapt task offloading and UAV paths at the same time, responding to changing IoT demands to keep total system delay as low as possible. This joint perspective is different from prior work that handles offloading and trajectory planning separately or relies only on heuristics.

In summary, the key contributions of this paper are:

(1) A collaborative computing framework for multiple UAVs in dynamic IoT settings: By allowing real-time task sharing and offloading among UAVs, the system can better handle uneven loads and sudden task surges than single-node or fixed-server setups.

(2) The integration of NOMA to enhance UAV swarm cooperation: With NOMA, multiple users can connect and offload tasks simultaneously, which improves spectrum utilization and throughput compared to traditional orthogonal access.

(3) A joint optimization design for task offloading and UAV trajectory planning: Task offloading and UAV trajectory planning are modeled together as a MDP, and a TD3-based algorithm helps UAVs adapt their actions dynamically to minimize total delay.

(4) Comprehensive simulations to verify the design: Various experiments show that the proposed solution achieves lower delays than traditional methods under different network loads and conditions, proving its practical value and scalability.

The remainder of this paper is organized as follows. Section 2 reviews related work and analyzes the current research gaps. Section 3 describes the system model, problem formulation, and the proposed joint optimization algorithm. Section 4 presents the simulation setup and discusses the results. Section 5 discusses the impact of each system component on overall performance and the expected practical implications of the proposed approach. Finally, Section 6 concludes the paper and outlines directions for future research.

## 2. Related Works 

In recent years, with the proliferation of Internet of Things (IoT) devices and mobile terminals, new mobile applications, such as real-time navigation, image processing, and video streaming, have increasingly posed important challenges for communication networks [4], including high costs and long time delays. These applications are often compute-intensive and latency-sensitive, which seriously tests the performance of traditional network architectures [6]. In order to address the above challenges, MEC, as an emerging distributed computing model, has been widely used to reduce the computing burden of terminal devices by deploying computing resources at the user’s proximal end, thereby improving network service efficiency and user experience [7,8]. Recent surveys, such as Javanmardi et al. [9], have further highlighted the importance of integrating security, privacy, and resource efficiency in IoT-Fog networks. However, these studies rarely address the specific challenges of multi-UAV collaborative MEC networks with high user concurrency and strict delay requirements. Although MEC can significantly improve the computing power of user equipment, it still faces the limitations of geographical environment and infrastructure construction during actual deployment, especially when the base station coverage is insufficient or the base station malfunctions, the stability and reliability of network services will be severely affected.

UAVs have become an ideal MEC auxiliary equipment due to its advantages of flexible mobility and rapid deployment. Especially in emergency situations, when the target area lacks effective base station support, UAVs can quickly fly to the target area to provide instant computing offloading and network coverage services. In the context of UAV-assisted MEC, a large number of related studies have been devoted to improving UAV service performance and network efficiency in recent years. Reference [10] presented a method that jointly optimizes resource allocation and task scheduling, aiming to reduce the weighted sum of UAV energy consumption and latency to improve the overall performance of UAV auxiliary services. Although the scheme performs well in a single UAV scenario, there remains a lack of sufficient in-depth research in the multi-UAV collaborative work scenarios. Tian et al. [11] proposed an optimization approach that jointly considers area partitioning and UAV trajectory planning to minimize the overall energy consumption of UAVs, with the objective of prolonging both flight duration and the operational lifespan of the network. However, the static scenarios assumed in their scheme are not suitable for complex dynamic environments, and it is difficult to cope with the rapid changes and unexpected situations of user needs, especially in the case of large-scale and multi-user collaboration, thus its adaptability and flexibility are limited. In addition, most of the above studies focus on the application scenarios of single-UAV-assisted computing, and fail to fully consider that a single UAV is difficult to meet the growing computing needs due to energy consumption and load limitations, which restricts the scalability and flexibility of UAVs in practical applications [12,13].

Therefore, with the gradual development of multi-UAV collaborative computing technology, more and more studies have begun to focus on the problem of UAV swarm collaboration, and explore how to solve the bottleneck of a single UAV in terms of computing power, load balancing, and delay control through multi-UAV collaboration, so as to improve the overall performance and service quality of the MEC system. Cheng et al. [14] formulated a joint optimization problem involving power control, task compression ratio, communication resource allocation, and UAV trajectory for wireless devices in a UAV-assisted edge computing data compression scenario. They employed an iterative block coordinate descent algorithm to reduce the overall energy consumption of the devices. In order to improve the computing power and response speed of the system, Zhuang et al. [15] studied the application of multi-UAV collaborative computing in edge computing, and proposed a multi-UAV collaboration strategy based on deep reinforcement learning to optimize computational offloading and flight paths. To improve the communication security in the multi-UAV scenario, Liu et al. [16] constructed an optimization problem of joint subcarrier allocation and UAV flight trajectory, and an iterative alternation algorithm with high computational efficiency was designed to solve the problem. Although the above study considers the multi-UAV environment, it mainly focuses on energy consumption optimization, fails to effectively combine the real-time requirements of latency and computing offloading, and has weak adaptability to the multi-user environment. In addition, Wu et al. [17] proposed a security-aware design for multi-UAV deployment, task offloading, and service placement using deep reinforcement learning to improve computational efficiency and system security. However, their work mainly focuses on security and service placement issues, rather than delay minimization in high-concurrency scenarios with NOMA. In order to solve the real-time problem, a multi-UAV collaborative task offloading scheme was developed in  [18] to optimize the UAV flight trajectory and task assignment to cope with the high latency requirements in the dynamic environment. However, there is no consideration for how to optimize latency in a multi-user environment. Sun et al. [19] introduced a collaborative task offloading strategy for multiple UAVs aiming to enhance offloading efficiency and minimize latency by jointly optimizing flight trajectory planning and computational resource allocation. Li et al. [20] focuses on the task offloading and flight trajectory optimization of multi-UAV cooperation, and adopts the strategy of joint power control and trajectory planning, with the goal of minimizing delay and improving system capacity. However, the above two schemes do not take into account the transmission queuing problem that may be caused by multi-user simultaneous upload tasks, which may affect the throughput and overall performance of the system in high-concurrency scenarios. In order to solve the problem of multi-user transmission queuing, Dehkordi et al. [21] considered the transmission queuing problem in UAV-assisted MEC system, and proposed a joint task offloading and transmission scheduling strategy to optimize the delay and system throughput. However, the DQN method used in the paper may have significant limitations in handling continuous action spaces, as it is prone to getting stuck in local optima and has poor adaptability to environmental changes. Wang et al. [22] proposed an optimization model based on queuing theory to reduce latency by rationally arranging task offloading and data transmission. Although this method is effective in a single-drone environment, it fails to fully consider the delay caused by multi-user simultaneous offloading tasks when it is extended to multi-UAV collaboration, and does not combine more efficient technologies (such as NOMA) to further optimize the delay. Reference [23] employed the Deep Deterministic Policy Gradient (DDPG) algorithm to coordinate and optimize the behavior of multi-UAV swarms. The authors introduced a central controller responsible for training on observed data and broadcasting the learned policy to the swarm network. However, the DDPG algorithm only considers the optimal action in the current state and requires pre-training, which limits its adaptability during real-time execution.

Although significant advancements have been achieved in areas such as computational capability enhancement, energy efficiency, and task offloading through multi-UAV collaboration, current research still exhibits notable limitations in effectively tackling delay optimization in multi-user scenarios. In particular, challenges related to transmission queuing and delay management under dynamic and time-varying conditions remain insufficiently addressed, leaving a gap in achieving truly responsive and efficient edge computing systems. Most existing solutions prioritize energy efficiency while neglecting the queuing delays caused by concurrent task offloading. Moreover, traditional optimization methods often struggle with large-scale, dynamic environments, demonstrating significant limitations in real-time performance and system adaptability.

To address these issues, NOMA technology provides a practical approach to enhancing spectrum efficiency and increasing system capacity. It effectively addresses resource contention in traditional wireless networks by allowing multiple users to communicate simultaneously over the same spectrum. Moreover, NOMA technology not only improves spectrum utilization but also minimizes user interference, thereby enhancing communication effectiveness and task offloading efficiency in multi-user scenarios.

Therefore, leveraging the advantages of Non-Orthogonal Multiple Access (NOMA) technology, this paper addresses the issues of transmission queuing and load imbalance in UAV-assisted collaborative computing by proposing a multi-UAV collaborative Mobile Edge Computing (MEC) network model integrated with NOMA. The proposed model is constructed for multi-UAV-assisted MEC scenarios, where tasks can be transferred among UAVs to achieve load balancing. Meanwhile, NOMA technology is introduced to alleviate transmission queuing caused by concurrent task uploads from multiple users. Taking into account both system latency and energy consumption constraints, this study formulates a comprehensive joint optimization problem that simultaneously addresses task offloading decisions and UAV trajectory planning, with the primary objective of minimizing total system delay. To effectively solve this complex problem, a tailored optimization approach based on the Twin Delayed Deep Deterministic Policy Gradient (TD3) algorithm is developed. By integrating the optimization of both task offloading strategies and UAV flight paths, the proposed framework achieves a further reduction in overall system delay, thereby enhancing the responsiveness and efficiency of the UAV-assisted edge computing environment.

From the above, it is clear that there is still a lack of practical models that integrate multi-UAV collaboration, NOMA-based access, and the joint optimization of task offloading and trajectory planning within a unified framework. Moreover, recent work by Zhang et al. [24] explored a digital twin-driven framework to enhance task offloading in UAV-assisted vehicle edge computing networks, indicating the potential of virtual representations for system awareness and adaptive control. Inspired by this, our work integrates NOMA and multi-UAV collaboration into a unified framework to address queuing delay and trajectory optimization more effectively.

## 3. Proposed Approach 

This section introduces the system architecture within the context of edge computing, along with the computational and communication models involved in the task offloading procedure. Table 1 summarizes the main symbols employed throughout the text, accompanied by their respective physical interpretations.

### 3.1. System Model

**Figure 1:**
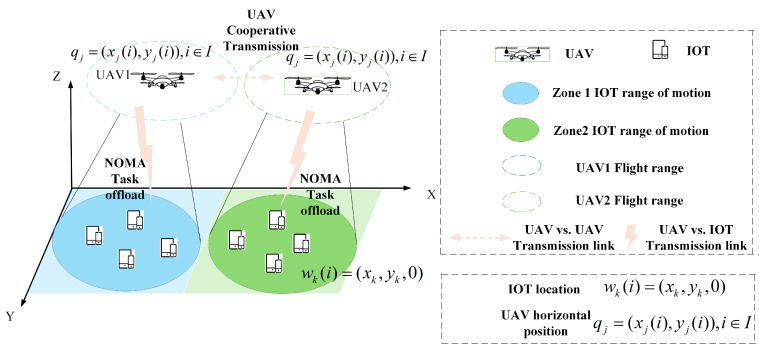
Model of multi-UAV collaborative edge computing system based on NOMA.

This study investigates a UAV-assisted mobile edge computing (UAV-assisted MEC) system consisting of several terminal devices and two UAVs, as shown in Figure 1. In this model, the entire area is divided into multiple subregions, with the classification of UAVs as either edge or cloud UAVs based on whether users within each region can directly offload tasks to the UAV. Specifically, the edge UAVs act as the edge layer of edge computing, responsible for providing computational services to IoT devices within their coverage area, while cloud UAVs, similar to cloud computing, are responsible for receiving tasks transmitted from other UAVs and performing computations. That is, each UAV can function as both an edge and a cloud UAV, receiving tasks from users within its own coverage area while also receiving tasks from other UAVs. This forms the collaborative principle of UAVs. To ensure flight safety and comply with relevant flight control policies, each UAV can only operate within a specified altitude range and is restricted by geographical boundaries, preventing it from crossing the defined area boundaries.

In this system, we consider a scenario without base stations, where *J* UAVs equipped with distributed micro-cloud servers are deployed across *J* regions. Each region contains one UAV and *K* IoT devices. For the sake of analysis, let r∈R={1,2,…,J} represent the set of divided regions, and $K_{r}=\left\{1,2,\ldots ,K\right\}$ represent the set of IoT devices within region r∈R. The UAV in region $J_{r}\in r$ is denoted as *j*, and the UAV in region $J_{o}\in r$ is denoted as *o*. In each time slot, the UAV in region *r* remains stationary and initiates a connection for communication with the IoT devices falling within its coverage zone. The IoT devices offload part of their tasks to the UAV using NOMA technology, which then performs computation and analysis. In order to reduce transmission queuing delay for IoT devices, UAVs in different regions can also transfer tasks to each other.

For the sake of convenience in discussion, the entire communication period *T* is discretized into *I* equal time slots $t_{i}\left(t_{i}=T/I\right)$, (i∈{1,2,…,I}). Meanwhile, a three-dimensional Cartesian coordinate system is adopted to represent the space, with the unit of measurement in meters (m).

The coordinates of IoT $k\in K_{r}=\left\{1,2,\ldots ,K\right\}$ in region *r* are denoted as $w_{k}\left(i\right)=\left(x_{k},y_{k},0\right)$. Assuming that all UAVs fly at a fixed safe altitude, the horizontal trajectory of UAV *j* in region *r* can be approximately represented as $q_{j}=\left(x_{j}\left(i\right),y\left(i\right)\right),i\in I$. Suppose $d_{j}\left(i\right)$ denotes the flight distance of the UAV during time slot *i*, and $\theta_{j}\left(i\right)$ represents the angle of the UAV’s horizontal direction relative to the positive X-axis in the XY plane. It is given that $0\leq d_{j}\left(i\right)\leq d_{\max}$, $0\leq \theta_{j}\left(i\right)\leq 2\pi$. Here, $d_{\max}$ stands for the theoretical maximum flight distance that the UAV can cover within a single time slot, and it must satisfy $d_{\max}=v_{max}I$.

Therefore, in each time slot, the flight trajectory along which the UAV moves is [25]:

(1) $$x_{j}\left(i\right)=x_{j}\left(0\right)+\sum_{i=1}^{I}d_{j}\left(i\right)\cos\theta_{j}\left(i\right)$$

(2) $$y_{j}\left(i\right)=y_{j}\left(0\right)+\sum_{i=1}^{I}d_{j}\left(i\right)\sin\theta_{j}\left(i\right)$$

where $d_{j}\left(i\right)=t_{fly}v\left(i\right)$, $t_{fly}$ represents the flight time, and v(i) represents the flight speed. Considering that the speed of the UAV remains constant, $v\left(i\right)=v_{0}t_{fly}$, where $v_{0}$ stands for the starting speed of the UAV. Moreover, the UAV can only fly within the specified area, which must satisfy $0\leq x_{j}\left(i\right)\leq x_{\max}$ and $0\leq y_{j}\left(i\right)\leq y_{\max}$, where $x_{\max}$ and $y_{\max}$ stand for the length and breadth of the region in sequence. Furthermore, due to the flight speed limitations, the flight path length of each UAV in any given time slot cannot exceed the maximum distance. Additionally, UAVs must maintain a minimum safe flight distance from each other to avoid collisions. Based on the above analysis, the mobility constraints that the UAV trajectories must satisfy are as follows:

(3) $$q_{j}\left(i+1\right)-q_{j}\left(i\right)\geq d_{\max}$$

(4) $$q_{j}\left(i\right)-q_{o}\left(i\right)\leq d_{\min}$$

where $d_{\min}$ represents the minimum safe distance between UAV *j* and UAV *o* within sub-region *r*. In this paper, each UAV is equipped with a mobile edge computing server and has communication relay capabilities. Therefore, a binary indicator $a_{j}^{o}\in \left\{0,1\right\}$ is used to represent whether UAV *j* and UAV *o* within sub-region *r* communicate. Let $a_{j}^{o}=1$ indicate that UAV *j* offloads data to UAV *o*, and $a_{j}^{o}=0$ indicate that UAV *j* does not offload data to UAV *o*. Then, we have

(5) $$a_{j}^{o}=\left\{\begin{array}{cc} 1, & \mathrm{if}\;\mathrm{UAV}\;i\;\mathrm{offloads}\;\mathrm{data}\;\mathrm{to}\;\mathrm{UAV}\;o,\\ 0, & \mathrm{if}\;\mathrm{UAV}\;i\;\mathrm{does}\;\mathrm{not}\;\mathrm{offload}\;\mathrm{data}\;\mathrm{to}\;\mathrm{UAV}o. \end{array}\right.$$

The IoT devices $k\in K_{r}=\left\{1,2,\ldots ,K\right\}$ move in a random manner while maintaining a constant speed within a flat region. In region *r*, the coordinates of IoT device *k* are indicated as $w_{k}\left(i\right)=\left(x_{k},y_{k},0\right)$. In each time slot, the IoT devices in region *r* transmit using NOMA technology to complete partial offloading of computation. In this paper, $a_{k}^{j}\left(i\right)\in \left\{0,1\right\}$ is used to indicate whether the near-end UAV provides computing services to IoT devices in time slot *i*. It is also assumed that the maximum number of tasks that each UAV can process simultaneously is $UAV_{\max}$. Therefore, the scheduling of IoT devices and the constraint on the number of tasks $UAV_{\max}$ for the UAV are as follows:

(6) $$a_{k}^{j}\left(i\right)\in \left\{0,1\right\}\;\forall i,k$$

(7) $$\sum_{j\in J_{r}}a_{k}^{j}\left(i\right)+\sum_{o\in J_{o}}a_{j}^{o}\leq UAV_{\max}$$

where $\sum_{j\in J_{r}}a_{j,o}^{k}$ represents the number of tasks transmitted by the IoT devices within the coverage area of this UAV in time slot *i*, and $\sum_{o\notin J_{o}}a_{j}^{o}$ represents the number of tasks transmitted to this UAV from other UAVs.

As mentioned earlier, the IoT devices within each region transmit tasks using NOMA technology. Therefore, the time-varying straight-line distance in Euclidean space between UAV *j* in region *r* and the IoT device is given by the following formula:

(8) $$d_{kj}\left(i\right)=\sqrt{H^{2}+{∥q_{j}\left(i\right)-\omega_{k}\left(i\right)∥}^{2}},\;0\leq i\leq I.$$

where *H* denotes the flight altitude of the UAV. In this paper, we maintain the UAV’s flight altitude at a fixed value. We assume that the communication link between the UAV and the ground IoT devices follows a Line-of-Sight (LoS) propagation model. Therefore, according to Shannon’s theorem, the UAV-related time-varying channel and the IoT devices within its coverage area can be described as:

(9) $$h_{k,j}\left(i\right)=\frac{\beta_{0}^{K}}{{\left(d_{k,j}\left(i\right)\right)}^{2}},\;0\leq i\leq I.$$

where $\beta_{0}^{K}$ signifies the channel gains at the reference distance of d=1 m. In this paper, tasks are transmitted between the IoT devices and UAVs using NOMA and Successive Interference Cancellation (SIC) technologies. According to Shannon’s theorem, the expression for the data transmission speed from the IoT device to the UAV is given by:

(10) $$R_{k,j}\left(i\right)=W_{k,j}\log_{2}\left(1+\frac{P_{k}h_{k,j}\left(i\right)}{\sum_{P_{k},h_{k^{\prime },j}\left(i\right)<h_{k,j}\left(i\right)P_{k}}h_{k^{\prime },j}\left(i\right)P_{k^{\prime }}+\sigma^{2}}\right)$$

where $\sum_{P_{k},h_{k^{\prime },j}\left(i\right)<h_{k,j}\left(i\right)P_{k}}h_{k^{\prime },j}\left(i\right)P_{k^{\prime }}$ represents the sum of the interference generated by devices smaller than device *k*, which is the product of the transmission power and the channel gain, $\sigma^{2}$ represents the Gaussian noise power, $W_{k,j}$ represents the channel bandwidth between the device and the UAV, and $p_{k}$ represents the transmission power between the device and the UAV. Similarly, the transmission rate between UAV *j* and UAV *o* is:

(11) $$\begin{array}{cc} R_{j,o}\left(i\right) & =W_{k,o}\log_{2}\left(1+\frac{P_{j}\rho }{{\left(d_{jo}\left(i\right)\right)}^{2}}\right)\\ \; & =W_{k,o}\log_{2}\left(1+\frac{P_{j}\rho }{H^{2}+{∥q_{j}\left(i\right)-q_{o}\left(i\right)∥}^{2}}\right) \end{array}$$

where $W_{k,o}$ represents the channel bandwidth between the UAVs, $d_{jo}\left(i\right)$ represents the time-varying Euclidean distance between UAV *j* and UAV *o*, and $P_{j}$ represents the transmission power between the UAVs. Here, $\rho =\frac{\beta_{0}^{J}G_{0}}{\sigma^{2}}$, with $G_{0}=2.2846$ [26,27,28] is a constant, and $\beta_{0}^{J}$ represents the channel gain when d=1 m during transmission.

In the UAV-assisted MEC framework proposed in this study, the data coming from each IoT device is processed by means of three components: local computation by the IoT device, edge UAV, and cloud UAV for auxiliary offloading and computation. Let $d_{kl}\left(i\right)$, $d_{ku}\left(i\right)$, and $d_{ko}\left(i\right)$ denote the offloading decisions of the UAVs for the IoT devices, where the constraint $d_{kl}\left(i\right)+d_{ku}\left(i\right)+d_{ko}\left(i\right)=1$ must be satisfied. As the volume of data which is returned to the IoT device subsequent to the processing of the task by the MEC server is trifling in comparison with the original data, the UAV offloading computation model expounded below will not consider the transmission delay and energy consumption regarding task return.

In this work, $M_{k}\left(i\right)=\left(F_{k}\left(i\right),D_{k}\left(i\right)\right)$ is used to represent the computational tasks originating from the IoT device in each time slot. Let $D_{k}\left(i\right)$ denote the size of the computational task in bits (bit), and $F_{k}\left(i\right)$ denote the number of the CPU cycles needed to fulfill the task. The relationship between $D_{k}\left(i\right)$ and $F_{k}\left(i\right)$ must satisfy $F_{k}\left(i\right)=C_{\mathrm{bit}}D_{k}\left(i\right)$. Here, $C_{\mathrm{bit}}$ signifies the number of CPU cycles which are necessary to process one unit of computational task.

When the IoT device processes the computational task locally, the local computation delay for the IoT device in time slot *i* is expressed as:

(12) $$T_{k}^{L}\left(i\right)=\frac{d_{kl}\left(i\right)F_{k}\left(i\right)}{f_{\mathrm{IoTD}}}$$

The IoT device offloads the computational task to the edge UAV. Based on the transmission rate discussed above, the transmission delay for the IoT device offloading the task to the UAV can be expressed as:

(13) $$T_{k}^{\mathrm{Tr}}\left(i\right)=\frac{d_{ku}\left(i\right)D_{k}\left(i\right)}{R_{k,j}\left(i\right)}$$

The computation delay required for processing tasks by the UAV is:

(14) $$T_{k}^{U}\left(i\right)=\frac{d_{ku}\left(i\right)F_{k}\left(i\right)}{f_{\mathrm{UAV}}}$$

where $f_{\mathrm{UAV}}$ represents the computational capacity of the UAV. The energy consumption produced by the UAV for computing tasks is:

(15) $$E_{k}^{U}=T_{k}^{U}\left(i\right)f_{\mathrm{UAV}}^{3}\gamma =d_{ku}\left(i\right)F_{k}\left(i\right)f_{\mathrm{UAV}}^{2}\gamma$$

In this case, $f_{\mathrm{UAV}}^{2}\gamma$ denotes the power consumption parameter decided by the effective charge, and γ denotes the factor that the chip structure impacts on the CPU’s computational capacity, typically valued at $10^{-27}$ [29].

The delay required for the task offloading process consists of both transmission delay and computation delay. Therefore, the total delay for offloading to the edge UAVs is:

(16) $$T_{k}^{O}\left(i\right)=T_{k}^{\mathrm{Tr}}\left(i\right)+T_{k}^{U}\left(i\right)$$

The IoT device offloads the computational task to the edge UAV, which then transmits it to the cloud UAV for computation. Based on the transmission rate discussed above, the transmission delay for the IoT device offloading the task to the cloud UAV is:

(17) $$T_{k}^{{\mathrm{Tr}}^{\prime }}\left(i\right)=\frac{d_{ko}\left(i\right)D_{k}\left(i\right)}{R_{k,j}\left(i\right)}+\frac{d_{ko}\left(i\right)D_{k}\left(i\right)}{R_{j,o}\left(i\right)}$$

The computation delay required for processing tasks by the cloud UAVs is:

(18) $$T_{k}^{U^{\prime }}\left(i\right)=\frac{d_{ko}\left(i\right)F_{k}\left(i\right)}{f_{\mathrm{UAV}}}$$

Similarly, the energy consumption generated by the remote UAV for computing tasks is:

(19) $$E_{k}^{U^{\prime }}=T_{k}^{U^{\prime }}\left(i\right)f_{\mathrm{UAV}}^{3}\gamma =d_{ko}\left(i\right)F_{k}\left(i\right)f_{\mathrm{UAV}}^{2}\gamma$$

The delay required for the task offloading process consists of the transmission delay and the computation delay. Therefore, the total delay for offloading to the cloud UAV is:

(20) $$T_{k}^{O^{\prime }}=T_{k}^{{\mathrm{Tr}}^{\prime }}\left(i\right)+T_{k}^{U^{\prime }}\left(i\right)$$

In summary, the delay consumed in each time slot within region *r* can be expressed as:

(21) $$T_{k}\left(i\right)=\max\left\{T_{k}^{L}\left(i\right),T_{k}^{O}\left(i\right),T_{k}^{O^{\prime }}\left(i\right)\right\}$$

Generally, the total energy consumption of a UAV can be categorized into three primary components: flight energy consumption, computation-related energy consumption, and hovering energy consumption. The flight energy consumption mainly depends on the UAV’s flight speed, flight distance, and hardware parameters. As described in [24], it is formulated as a function of the UAV’s mass $M_{\mathrm{UAV}}$, expressed through the following value function:

(22) $$E_{\mathrm{fly}}\left(i\right)=0.5M_{\mathrm{UAV}}t_{\mathrm{fly}}v{\left(i\right)}^{2}$$

In this paper, we do not take into account the energy consumption of UAVs during hovering  [30]. Consequently, the total energy expenditure of a UAV in region *r* is expressed as:

(23) $$E_{\mathrm{UAV}}=E_{\mathrm{fly}}\left(i\right)+E_{k}^{U}\left(i\right)+E_{k}^{U^{\prime }}\left(i\right)$$

To guarantee the required quality of service, the total energy consumed by the UAV in executing all assigned computational tasks must remain within the limits of its initial battery capacity: $\sum_{i=1}^{I}E_{\mathrm{UAV}}\left(i\right)\leq E_{\mathrm{UAV}}$.

### 3.2. Problem Statement

In this paper, our objective is to minimize the system latency, subject to the constraints associated with discrete variables and energy consumption. To achieve this, NOMA technology is employed, coupled with the joint optimization of UAV trajectories and task offloading ratios. In the current research, let the pair $U≜\{d_{j}\left(i\right),\theta_{j}\left(i\right),\forall i\in I\}$ be used to characterize the UAV’s flight trajectory over time, while $A≜\{a_{kl}\left(i\right),a_{ku}\left(i\right),a_{ko}\left(i\right),\forall i\in I\}$ is employed to denote the offloading decision variables corresponding to the IoT device. Each IoT device in a region can simultaneously perform local computation and task offloading. Therefore, the total delay for the entire system to complete all tasks depends on the maximum value of the time taken to complete local computation, edge computation, and remote computation in each region. Thus, the total delay for the entire system to complete all tasks is:

(24) $$T^{D}=\sum_{i\in I}\max\left\{\sum_{k=1}^{K}T_{k}^{L}\left(i\right),\sum_{k=1}^{K}T_{k}^{O}\left(i\right),\sum_{k=1}^{K}T_{k}^{O^{\prime }}\left(i\right)\right\}$$

The specific optimization problem is modeled as:

(25) $$\min_{A,U}T^{D}$$

Subject to:

(26) $$\left.\begin{array}{cc} \; & C1:a_{k}^{o}\left(i\right)\in \left\{0,1\right\},\;\forall i,k,\\ \; & C2:a_{k}^{l}\left(i\right)\in \left\{0,1\right\},\;\forall i,k,\\ \; & C3:a_{k}^{u}\left(i\right)\in \left\{0,1\right\},\;\forall i,k,\\ \; & C4:a_{k}^{o}\left(i\right)+a_{k}^{l}\left(i\right)+a_{k}^{u}\left(i\right)=1,\;\forall i,k,\\ \; & C5:0\leq d_{j}\left(i\right)\leq d_{\max},\;\forall i,j,\\ \; & C6:0\leq \theta_{j}\left(i\right)\leq 2\pi ,\;\forall i,j,\\ \; & C7:0\leq x_{j}\left(i\right)\leq x_{\max},\;\forall i,j,\\ \; & C8:0\leq y_{j}\left(i\right)\leq y_{\max},\;\forall i,j,\\ \; & C9:\;∥q_{j}\left(i+1\right)-q_{j}\left(i\right)∥\;\leq d_{\max},\;\forall i,j,\\ \; & C10:\;∥q_{j}\left(i\right)-q_{o}\left(i\right)∥\;\geq d_{\min},\;\forall i,j,\\ \; & C11:\sum_{i=1}^{I}E_{\mathrm{UAV}}\left(i\right)\leq E_{\mathrm{UAV}},\\ \; & C12:\sum_{i=1}^{I}\sum_{k=1}^{K}D_{k}\left(i\right)=D_{\mathrm{total}}. \end{array}\right\}$$

wherein, the constraint C1 indicates that the IoT device performs local computation and decision-making. C2 represents the computation and decision-making offloading to the proximal drone, while C3 represents the computation and decision-making offloading to the distal drone. C4 ensures that the computation tasks of the IoT device consist of local computation, proximal drone offloading, and distal drone offloading, and establishes constraints on the decision relationships among these three parts of the computational tasks to ensure reasonable task allocation. Constraints C5 and C6 are associated with the flight distance and angle of the drone. C7 and C8 represent the flight range constraints for the drone, ensuring that its flight position remains within the allowed range. C9 ensures that the flight distance of the drone during any time slot does not exceed the theoretical maximum value. Constraint C10 is designed to avoid collisions during flight, while C11 accounts for the drone’s energy constraints. Finally, C12 ensures that all tasks are completed within the entire task cycle, imposing the constraint that the system must finish all tasks.

In summary, there are non-convex restrictions and objective functions in this paper, and the optimization variables are mutually associated. Traditional optimization methods are often constrained by their dependence on the initial solution, which limits their ability to thoroughly explore the entire search space and typically leads to suboptimal results. In addition, these approaches tend to struggle with complex and dynamic environments, particularly when faced with continuous action spaces, where their performance becomes significantly limited [24,30]. Although DDPG has improved in dealing with continuous action space, it is prone to Q overestimation and instability during training. The TD3 algorithm effectively solves the challenges of traditional optimization algorithms and DDPG by introducing double delayed updates, target strategy smoothing, and noise processing, and provides more stable and accurate optimization results, especially in continuous action space problems such as task offloading and trajectory optimization, showing stronger performance and robustness [31].

### 3.3. Proposed TD3-Based Optimization Approach

TD3 is an advanced deep reinforcement learning algorithm, which is an extension of the deterministic policy gradient method. Compared with the traditional algorithm, TD3 significantly improves the stability and performance of the algorithm through the double Q learning mechanism, delayed policy update and target strategy smoothing, so that it can more efficiently cope with the optimization problem in the continuous action space. The TD3 algorithm can be used to optimize the delay problem, which can effectively enhance the global search ability of task offloading decisions, improve the convergence speed and adaptability of the algorithm, and make it better suited for task offloading as well as trajectory optimization in complex dynamic environments. To address this challenge, this study introduces a TD3-based algorithm aimed at minimizing latency in multi-user environments by jointly optimizing the UAV’s flight trajectory and the task offloading strategies of ground IoT devices. Based on the MDP model, the algorithm uses the state-action-reward quadruple as the training sample to optimize the trajectory of the drone instantaneously and determine the optimal task offloading ratio, so as to reduce the delay.

To enable the application of the TD3 algorithm to the optimization task, the original problem is reformulated within an MDP framework. Within this structure, the state space, action space, and reward function of the computation offloading problem are defined as follows:

State space:

(27) $$s_{i}^{r}=\left\{E_{\mathrm{battery}}\left(i\right),D_{\mathrm{remain}}\left(i\right),q_{j}\left(i\right),\omega_{k}\left(i\right),D_{k}\left(i\right)\right\}$$

In the equation, $E_{\mathrm{battery}}$ represents the remaining battery of the UAV, $D_{\mathrm{remain}}\left(i\right)$ represents the size of the remaining task, and it must satisfy $D_{\mathrm{remain}}\left(i\right)=D-\sum_{i=1}^{I}\sum_{k=1}^{K}D_{k}\left(i\right)$. Where i=1, $D_{\mathrm{remain}}\left(i\right)=D$, and $E_{\mathrm{battery}}\left(i\right)=E_{\mathrm{UAV}}$.

Action space: The action space in this article is defined as the proportion of the task offloading and the flight trajectory of the drone. Therefore, the action space is:

(28) $$a_{r}^{i}=\left\{d_{kl}\left(i\right),d_{ku}\left(i\right),d_{ko}\left(i\right),d_{j}\left(i\right),\theta_{j}\left(i\right)|\forall k\in K,\forall i\in I\right\}$$

Reward function: The primary objective of this study is to minimize task execution delay as effectively as possible. Since each selected action is directed toward reducing latency, the reward function is accordingly defined to reflect this goal and can be formulated as follows:

(29) $$r_{i}^{r}=r\left(s_{i}^{r},a_{i}^{r}\right)$$

Building upon the MDP formulation outlined in Section 4.1, this paper proposes a task offloading and trajectory optimization algorithm based on the TD3 framework. The algorithm is specifically tailored to effectively handle the high-dimensional and continuous nature of the action space inherent in the task offloading optimization problem. The TD3 algorithm is a deterministic deep reinforcement learning method based on the Actor-Critic framework, which improves the stability and learning effect of the algorithm by improving the Critic network structure and update mechanism. The TD3 algorithm employs a dual-network framework composed of the main network and the target network. In this setup, each of these networks encompasses an actor network along with two Critic networks, specifically designated as Critic1 and Critic2. The target network plays a crucial role in generating steady target values, which serves to enhance the stability of the learning process. Meanwhile, the main network takes on the responsibility of conducting real-time policy evaluation and implementing updates.

Among them, the Actor network generates specific action strategies based on the state inputs of the environment, optimized with the goal of maximizing future cumulative returns, and its parameters are updated as shown in Equation (34). The two Critic networks receive the state and action as input, evaluate the quality of the current policy, and output the value of the state-action pair. In order to alleviate the problem of overestimation of Q value in the traditional method, TD3 adopts the double-Q network mechanism, and updates the target value by selecting the smaller value of the output value of Critic1 and Critic2 networks, and the target value is calculated as shown in Equation (32). The Critic network parameters are updated by reducing the error between the predicted and target values, as detailed in (33).

To enhance the stability of the target value, the TD3 algorithm incorporates a smoothing regularization mechanism during its computation. This is achieved by introducing controlled random noise to the target action value, which helps to mitigate abrupt fluctuations. The resulting adjusted target action value is presented in Equation (31). The elements of the target network which act as parameters are adjusted step by step by the soft update method, and the update rules are shown in Equation (35), so as to ensure the smoothness and reliability of the output throughout the training process.

Additionally, the TD3 algorithm adopts an experience replay mechanism. The interaction data generated between the actor network and the environment is stored as experience tuples in a replay buffer. During training, small batches are randomly sampled from this buffer to update the network parameters, promoting learning stability and efficiency. This approach not only effectively lessens the dependence and correlation among samples, but also significantly improves the convergence speed and stability of the algorithm.

**Figure 2:**
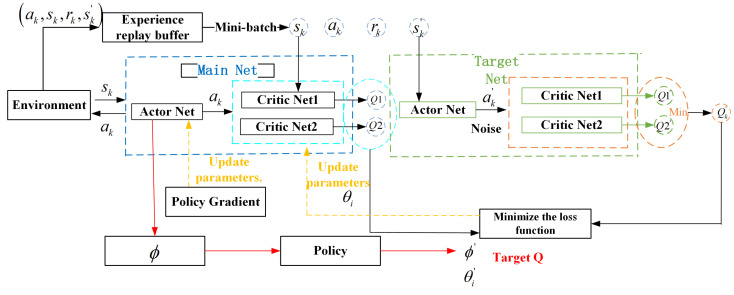
TD3 algorithm framework.

Therefore, the TD3 algorithm improves the accuracy of Q value calculation through the double-Q network architecture, and significantly enhances the stability and performance of the algorithm by combining the delayed update mechanism and target value smoothing. Figure 2 illustrates the framework diagram of the TD3 algorithm.

First, the network parameters are initialized, and then the initial state is input into the Actor network. In each iteration, the drone selects action *a* based on the Actor network $\pi_{\phi }\left(s\right)$ and random noise ϵ, and after performing the current action, it gets a reward $r_{k}$ and the state $s_{k+1}$ of the next moment, so it gets a quadruple $\left(s_{k},a_{k},r_{k},s_{k}^{\prime }\right)$. Then the quadruple is stored in the empirical buffer *R*, when the buffer space is stored, *N* tuples will be randomly sampled in the replay pool, assuming that the data we sample is $\left(s_{k},a_{k},r_{k},s_{k}^{\prime }\right)$, then the action of the state $s_{k}^{\prime }$ is:

(30) $$a_{k}^{\prime }=\pi_{\theta^{\prime }}\left(s_{k}^{\prime }\right)$$

Then, based on the target network policy smoothing regularization, random noise is added to the Actor network, then the modified target action value is:

(31) $$a=\pi_{\theta^{\prime }}\left(s_{k}^{\prime }\right)+\epsilon :\epsilon ∼N\left(0,\sigma^{2}\right)$$

Then, based on the idea of a dual network, the goals of the Critic network are defined as:

(32) $$y=r+\min_{i=1,2}\left\{Q_{\theta_{i}}^{\prime }\left(s_{k}^{\prime },\tilde{a}\right)\right\}$$

The parameter $\theta_{i}$ of the two Critic networks is then updated by minimizing the loss function:

(33) $$LossFunction=\frac{1}{B_{s}}\sum {\left(Q_{\theta_{i}}\left(s_{k},a_{k}\right)-y\right)}^{2}$$

Then, combined with the Q function of the Critic training network, the policy gradient of the actor when the parameters are updated can be obtained:

(34) $$\nabla_{\phi }J\left(\phi \right)=\frac{1}{B_{s}}\sum \nabla_{a}Q_{\theta_{i}}{\left(s,a\right)|}_{a=\pi_{\phi }\left(s\right)}\nabla_{\phi }\pi_{\phi }\left(s\right)$$

Finally, update target networks $\theta_{i}^{\prime }$ and $\phi^{\prime }$:

(35) $$\left\{\begin{array}{cc} \; & \theta_{i}^{\prime }\leftarrow \tau \theta_{i}+\left(1-\tau \right)\theta_{i}^{\prime },\\ \; & \phi^{\prime }\leftarrow \tau \phi +\left(1-\tau \right)\phi^{\prime } \end{array}\right.$$

In the optimal task offloading decision-making process based on the TD3 algorithm proposed in this paper, the Actor and Critic network parameters are updated iteratively. Simultaneously, the UAV’s flight trajectory and task offloading strategy are refined to minimize delay. Algorithm 1 outlines the algorithm flow. Initially, the network framework is set up, including random parameter initialization for the Critic and Actor networks, along with their respective target networks. An experience buffer is then established to store interaction data. The iterative training process follows, with lines 1 to 18 forming the complete training procedure. In each iteration, the system first initializes the environment, receives the state $s_{0}$, and selects action $a_{i}$ according to the current strategy and Gaussian noise, which includes the flight direction, flight distance and task offloading decision of the drone. After the action is executed, the system calculates the reward $r_{i}$ and the next state $s_{i+1}$, and deposits the generated quadruple $\left(s_{i},a_{i},r_{i},s_{i+1}\right)$ into the empirical buffer *R*, as shown in line 9. Lines 10 to 15 involve randomly sampling mini-batches of empirical data from the buffer and adjusting the network parameters based on Equations (33)–(35). This process is iteratively carried out until the maximum number of steps, Max_Step, is achieved. Ultimately, the algorithm generates the optimal UAV flight path, task offloading strategy, and minimizes the system’s delay. Algorithm 1 details the implementation of the optimal task offloading strategy using the TD3 framework.

In this study, the TD3 algorithm is used to optimize the offloading decisions of multi-UAV collaborative computing tasks and the selection of flight trajectories. The computational complexity of the neural network is influenced by various factors, including the size of the data, the complexity of the model, and the overall algorithmic framework. To simplify this, we focus on analyzing the computational complexity of generating the optimal action. In each iteration, each agent in TD3 traverses all actions to find the optimal action with the maximum Q-value. In the system model proposed in this paper, there are *K* IoT devices and *J* UAVs in each time slot. The offloading decision of each IoT and the flight trajectory of each UAV together form an action space, denoted as $M_{total}=K\cdot N$, where *N* refers to the number of offloading decisions for each IoT device. Therefore, the total action space size is $M_{total}$. In each iteration, the TD3 algorithm selects the optimal action by having the agent traverse the action space. Thus, the computational complexity is $O(T\times J\times M_{total})$, with *T* representing the iteration period. That is, the overall complexity is O(T×J×N×K).

**Algorithm 1** Optimal Task Offloading Decision Algorithm Based on TD3**Input:** number of iterations, learning rate for the Actor network, learning rate for theCritic network, discount factor, and soft update coefficient**Output:** offloading decisions, UAV trajectory, and minimum delay1Randomly initialize parameters $\theta_{1}$, $\theta_{2}$ for the Critic networks; randomly initialize parameter ϕ for the Actor network2Initialize target networks: $\theta_{1}^{\prime }\leftarrow \theta_{1}$, $\theta_{2}^{\prime }\leftarrow \theta_{2}$, $\phi^{\prime }\leftarrow \phi$3Initialize the experience replay buffer: *R*4**for** episode =0 to Max_Step **do**5   Reset environment and observe initial state $s_{0}$6   **for** time step t=1 to *T* **do**7      Select action $a_{t}=\pi_{\phi }\left(s_{t}\right)+\epsilon$, ϵ represents Gaussian noise8      Execute $a_{t}$, the reward $r_{t}$ and the next state $s_{t+1}$ are obtained9      Store its data tuple $\left(s_{t},a_{t},r_{t},s_{t+1}\right)$ into the experience buffer *R*10      if *R* is full, update the experience buffer11      Randomly sample a batch of *N* values from the multidimensional array
$\left(s_{i},a_{i},r_{i},s_{i+1}\right)$, from i=1,2,…,N12      Update the Critic network by minimizing the target loss using Equation (33)13      **if** kmodd==0 **then**14         Update the Actor online network using Equation (34)15         Update the weights of the target network according to Equation (35)16      **end if**17   **end for**18
**end for**


## 4. Performance Evaluation 

### 4.1. Simulation Setup 

This section conducts a performance evaluation of the proposed TD3-based scheme for task offloading and trajectory optimization in a multi-UAV-assisted edge computing environment, with an emphasis on reducing overall system delay. The experimental setup was implemented using Python 3.8 and the TensorFlow framework, running on a PC powered by an AMD Ryzen 7 4800U processor (AMD, Santa Clara, CA, USA). In the experiment, we deployed two UAVs and eight IoT devices. The IoT devices are randomly deployed across two adjacent rectangular regions. Area 1 covers a 200 m × 200 m square, while Area 2—an equally sized 200 m × 200 m region—is positioned adjacent to Area 1 along the X-axis, spanning coordinates from 200 m to 400 m. Thus, the X-coordinate range for Area 2 is defined as (200 m, 400 m), with both areas sharing identical Y-axis dimensions.

#### 4.1.1. Simulation Metrics

To evaluate the performance of the proposed approach, comparative experiments were conducted using the DDPG algorithm [32], the Deep Q-Network (DQN) algorithm [33], the Actor-Critic (AC) algorithm [34], the UAV no-coordination scheme, and local processing schemes under the same system model environment. The evaluation indicators include the convergence of the algorithm in this scheme and the cost when using different algorithms to process the same task. To provide an objective comparison of various solutions, the evaluation focused on five key aspects: task volume, number of IoT devices, flight speed, flight duration, IoT computing capacity, and UAV computing capacity.

#### 4.1.2. Simulation Scenarios

The UAVs maintain a fixed altitude within their respective areas, covering their designated service ranges and assisting in task offloading. The task size was configured at 80 Mbits, while the computing capacities of the UAV and IoT devices were set to 1.2 GHz and 0.6 GHz, respectively. Additional parameters can be found in Table 2, as detailed in Refs. [35,36]. The experiments involved running multiple iterations of Algorithm 1 under identical conditions. Finally, the collected data were systematically analyzed for performance comparison.

### 4.2. Experimental Results

To gain a deeper understanding of the algorithm’s performance and stability, we conducted a detailed analysis of the key hyperparameters during the experimental process. This includes adjustments to memory capacity MEMORY_CAPACITY, learning rate $\alpha_{a},\alpha_{c}$, and discount factor γ. By comparing the relationship between the number of training iterations and cumulative rewards under different hyperparameter configurations, we ultimately selected the optimal parameter combination that balances training stability and convergence efficiency. It is important to note that the reward used in this paper is the negative value of the total system delay, which serves as the agent’s feedback signal in reinforcement learning to guide policy optimization. Although this reward is constructed based on delay with physical units, within the reinforcement learning framework, it essentially serves as a relative measure of policy quality and does not retain the original physical units.

**Figure 3:**
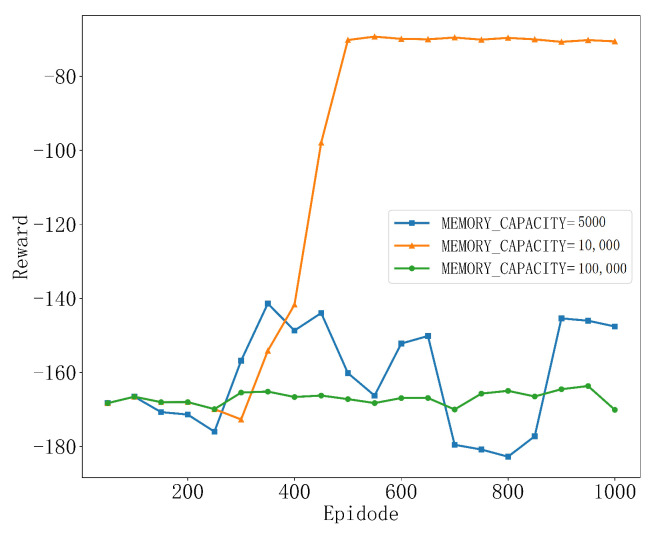
Convergence performance at different memory capacities.

As shown in Figure 3, the best performance is achieved when the memory capacity is set to MEMORY_CAPACITY = 10,000. This is because, at this setting, the replay pool can store more samples, allowing the training process to learn from a more diverse set of experiences, which helps the algorithm find a more stable convergence path. However, when the memory capacity is set too large, such as MEMORY_CAPACITY = 100,000, the reward variation throughout the learning process is small, with almost no significant fluctuations, showing an overly smooth trend. This may be due to the excessively large memory capacity causing the agent to overfit the data in the training environment, preventing it from learning more generalized strategies. When the memory capacity is set to MEMORY_CAPACITY = 5000, we observe significant fluctuations in the agent’s learning process, and it ultimately fails to converge. This is because with a smaller memory capacity, the experience replay pool used during training may not provide enough diverse samples, leading to overly similar experiences being sampled during training, which can cause overfitting or unstable convergence. Therefore, a moderate memory capacity usually yields the best results, and in this chapter, the memory capacity is set to MEMORY_CAPACITY = 10,000.

**Figure 4:**
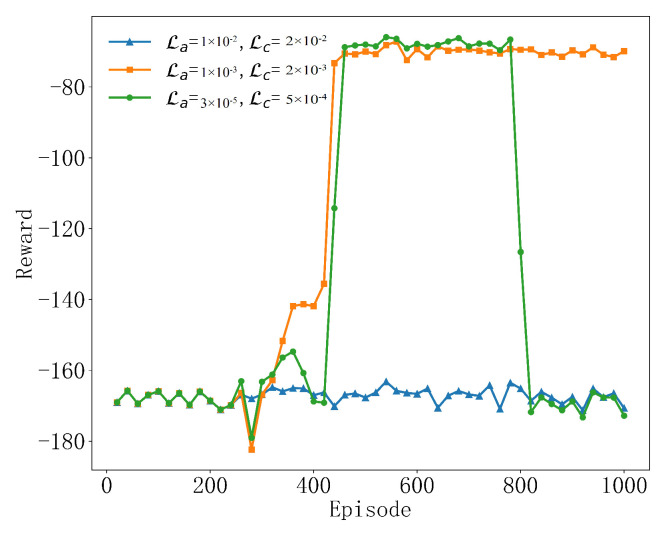
Convergence performance at different learning rates.

As shown in Figure 4, the selection of the learning rate plays a significant role in determining the agent’s learning efficiency and strategic performance. When the learning rate is $\alpha_{a}=1\times 10^{-3}$, $\alpha_{c}=2\times 10^{-3}$, it shows the best performance among the three settings, and the reward value experiences a rapid increase in the initial stage and quickly converges to a high stable value after about 400 rounds. This indicates that this learning rate configuration achieves a good balance between exploration and utilization, so that the agent can learn and optimize the strategy efficiently. When the learning rate is small, $\alpha_{a}=3\times 10^{-5}$, $\alpha_{c}=5\times 10^{-4}$, the region is stable at 400 to 800 rounds, but after 800 rounds, it shows large fluctuations and the reward is very reduced, which indicates that the algorithm may have difficulty finding effective strategies at low learning and exploration rates. Although it stabilized in the medium term, the overall performance was not as good as $\alpha_{a}=1\times 10^{-3}$, $\alpha_{c}=2\times 10^{-3}$. When the learning rate is excessively high, $\alpha_{a}=1\times 10^{-2}$, $\alpha_{c}=2\times 10^{-2}$, the reward value of this setting is basically stable around −170. This may indicate that the high learning and exploration rates have led to the algorithm’s exploration being too aggressive and may not have made full use of what has been learned to optimize its decision-making, resulting in an average performance. Therefore, this paper chooses to set the learning rate to $\alpha_{a}=1\times 10^{-3}$, $\alpha_{c}=2\times 10^{-3}$, which supports the trade-off between effective learning and stable performance of the algorithm, so that it gradually converges to a better strategy during the training process, effectively minimizing the negative value of the reward, that is, maximizing the efficiency. These results show that moderate learning and exploration rates are essential for the success of deep reinforcement learning algorithms.

**Figure 5:**
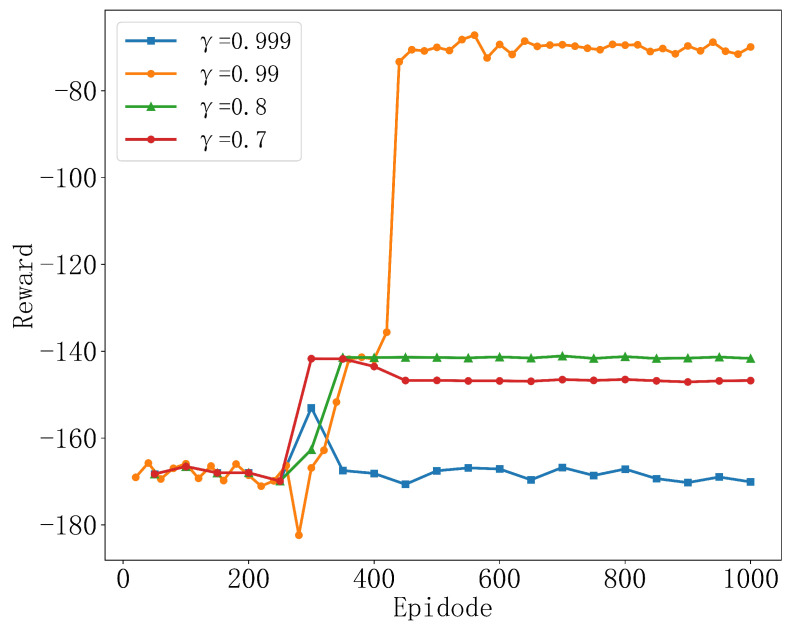
Convergence performance at different discount factors.

Figure 5 shows the variation in rewards of the TD3 algorithm under different discount factor γ values. The simulation results clearly indicate that the choice of discount factor γ=0.99 performs the best in the simulation. This reflects that, when considering long-term strategies, the TD3 algorithm effectively balances immediate and future rewards, enabling the agent to develop a more comprehensive and optimized behavior strategy, ultimately achieving higher cumulative rewards. Compared to other choices of discount factors, such as γ=0.7 and γ=0.8, although they show stability in rewards, their overall performance is lower than that of γ=0.99. This is because, compared to γ=0.99, these values place less weight on long-term rewards. The algorithm tends to be more conservative when considering future rewards, placing more focus on short-term rewards, which may lead the model to rely too heavily on immediate rewards during the learning process, neglecting long-term planning. This is reflected in the fact that the reward value shows little to no change throughout the entire training process, which could be due to the specific settings of the TD3 algorithm at this discount rate not being suitable for this environment, leading to a lack of motivation in the learning process to explore better solutions. Experimental results show that an appropriately high discount factor helps seek optimal long-term strategies in complex decision-making environments. Therefore, in this chapter, the discount factor is set to γ=0.99.

**Figure 6:**
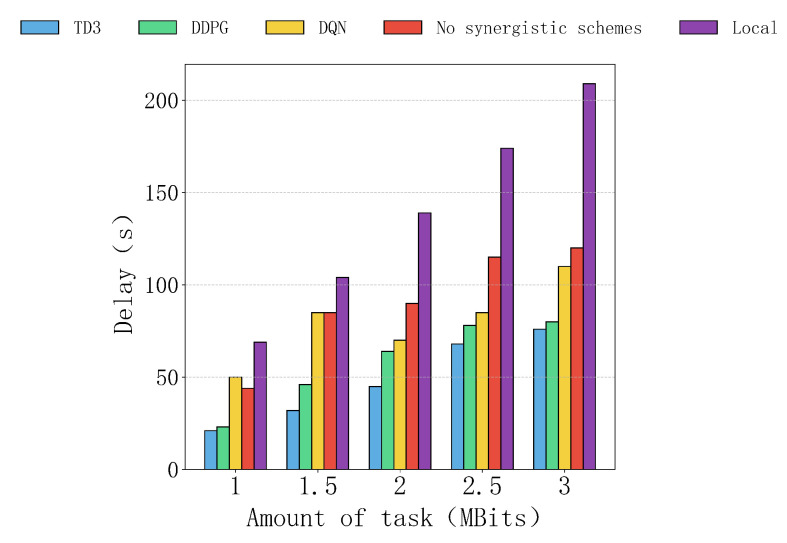
The impact of different task sizes on delay.

Figure 6 shows the delay of five computational schemes with task sizes ranging from 1 MBit to 3 MBits. Overall, the TD3 algorithm consistently performs the best, with the lowest delay and slowest growth, while local computation has the highest delay, which increases rapidly in a linear fashion as the task size increases. The delays of other schemes fall between these two extremes, with DDPG performing second best. DQN remains competitive at smaller task sizes, but its performance deteriorates significantly as the task size grows. The non-collaborative scheme shows the lowest overall efficiency.

In terms of specific performance, the TD3 algorithm has a clear advantage at any task size, indicating that it is the most efficient in task allocation and resource utilization, able to handle tasks of varying sizes stably. The DDPG algorithm performs similarly to TD3 at a task size of 2 MBits but slightly lags behind at higher task sizes. DQN performs decently at a task size of 1 MBit, but as the task size increases, its delay grows significantly, indicating poor resource utilization efficiency. The delays of the non-collaborative scheme and local computation are much higher than those of other collaborative schemes. The non-collaborative scheme relies entirely on the local edge server, which leads to idle remote UAVs and low efficiency. Local computation, relying solely on the device’s own resources, performs particularly poorly at larger task sizes, with delays significantly higher than other schemes.

**Figure 7:**
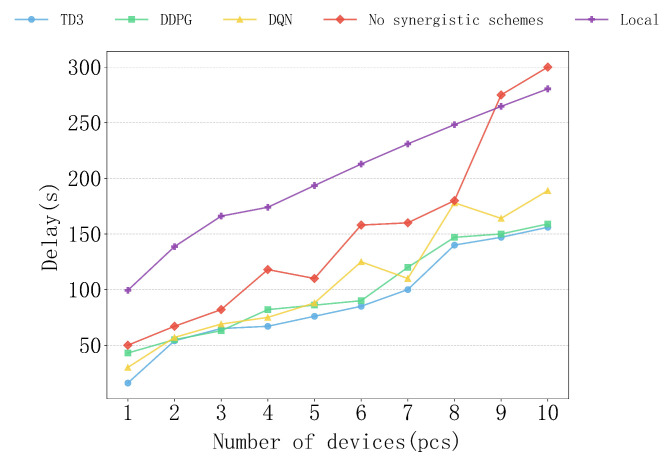
The impact of different device numbers on delay.

Figure 7 shows the delay performance of various algorithms under different scenarios with the number of devices ranging from 1 to 10. From the results, it can be observed that the TD3 algorithm consistently demonstrates the best delay control capability, with the delay growth being the most gradual, exhibiting significant performance advantages. In contrast, the DDPG and DQN algorithms perform second best, while the non-collaborative and local computation schemes have significantly higher delays than the other algorithms, with a steeper growth trend.

Upon analysis, it can be seen that the TD3 algorithm performs particularly well when the number of devices is high, such as 8–10 devices. Its excellent resource allocation and collaborative computing capabilities keep the delay at a lower level, demonstrating strong stability and adaptability. The DDPG algorithm performs similarly to TD3 with fewer devices but shows a slightly faster delay growth as the number of devices increases, with performance slightly lagging behind TD3. The DQN algorithm performs decently with 1–3 devices but experiences a rapid increase in delay as the number of devices grows, showing unstable performance. The delays of the non-collaborative and local computation schemes remain high throughout, especially the local computation scheme, where the delay almost increases linearly as the number of devices grows, resulting in the lowest efficiency. Therefore, the TD3 algorithm demonstrates the best delay optimization ability in the research environment.

**Figure 8:**
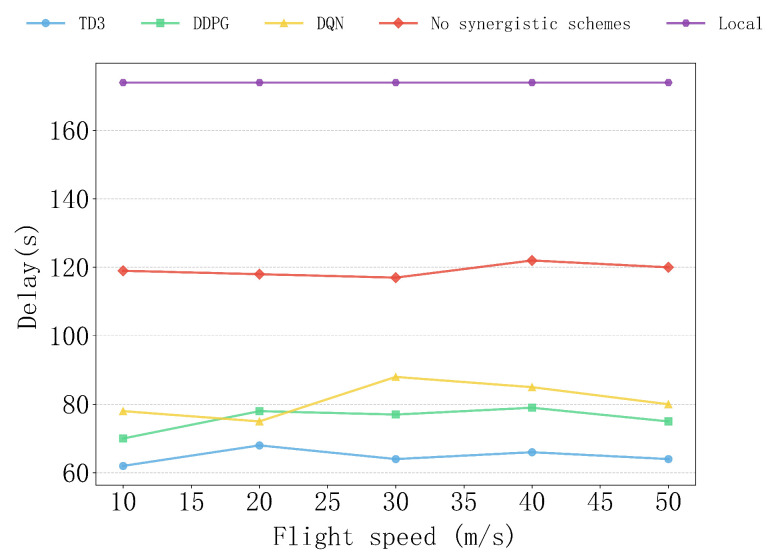
The impact of different flight speeds on delay.

Figure 8 shows the delay performance of various algorithms, as well as the non-collaborative and local computation schemes, under different flight speeds. Overall, the TD3 algorithm consistently maintains the lowest delay at all flight speeds. In contrast, the DDPG algorithm performs similarly to TD3 at low speeds (10–20 m/s), but shows slight fluctuations and a slight increase in delay when the speed increases to 30–40 m/s. The performance of the DQN algorithm is relatively significant, with its delay peaking at speeds of 20–30 m/s, followed by a slight decrease. This may be due to the algorithm’s limited flexibility in resource scheduling.

The delays of the non-collaborative and local computation schemes are always higher than those of the other algorithms, especially the local computation scheme, where the delay remains at the highest level and is completely unaffected by the flight speed. This is because the local computation scheme relies entirely on the user device for computation, so the delay depends solely on the device’s computing performance and is independent of the flight speed. As a result, the delay follows a steady high-level curve, consistently higher than the other schemes. The non-collaborative scheme, relying entirely on the user device and UAV to perform computations independently without collaboration, has relatively stable and high delays. Therefore, compared to the other schemes, the TD3 algorithm is more efficient in adapting to changes in flight speed, fully demonstrating its superior performance in handling multi-task problems in dynamic environments.

**Figure 9:**
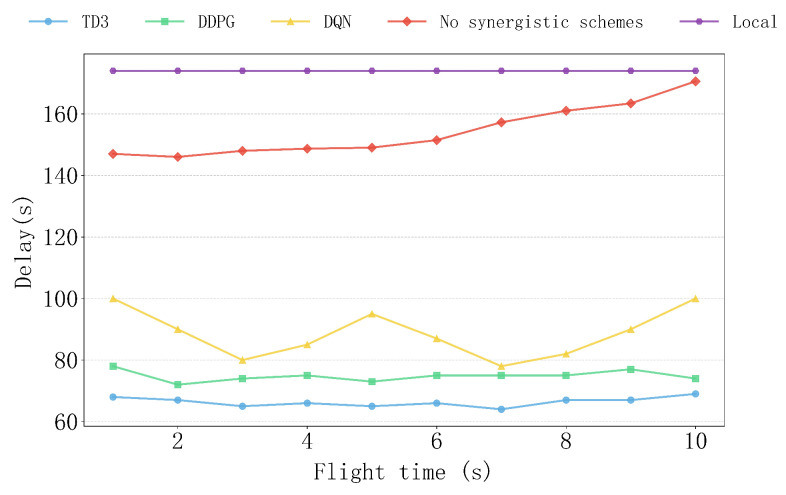
The impact of different flight time on delay.

Figure 9 shows the delay performance of various algorithms, as well as the non-collaborative and local computation schemes, under different flight times. The TD3 algorithm demonstrates extremely high stability with changing flight times, with its delay curve being smooth and the lowest, significantly outperforming other algorithms. In contrast, although the DDPG algorithm maintains relatively stable delay performance as the flight time increases, it is still slightly inferior to TD3. The delay of the DQN algorithm is relatively higher and shows some fluctuations, which is due to the inherent discreteness of the DQN algorithm, leading to less adaptability in continuous dynamic environments. The delays of the non-collaborative and local computation schemes remain consistently high. The delay of the non-collaborative scheme increases gradually as the flight time increases, mainly due to the prolonged UAV flight time, which exacerbates communication delays and accumulates task processing time.

**Figure 10:**
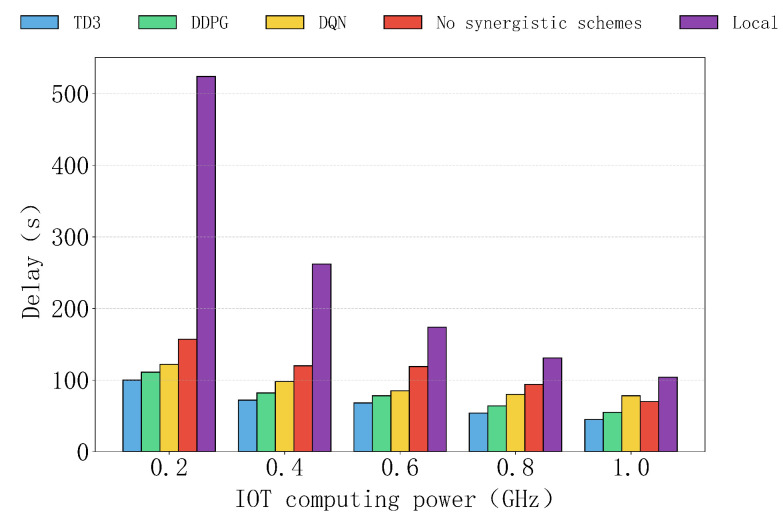
The impact of computing power of different IOTs on delay.

Figure 10 shows the delay performance of various algorithms, as well as the non-collaborative and local computation schemes, under different device computation capabilities. Overall, the TD3 algorithm consistently exhibits the lowest delay under all device computation capabilities, significantly outperforming other algorithms and demonstrating its excellent task scheduling performance. In contrast, the delays of the DDPG and DQN algorithms are relatively higher but still maintain a low level, especially at higher computation capabilities, around 0.6 GHz, where their performance is close to that of TD3.

The delays of the non-collaborative and local computation schemes remain consistently high, with the local computation scheme experiencing a sharp increase in delay at lower computation capabilities, such as 0.2 GHz. This is because, for the local computation scheme, as the task depends entirely on the device’s computation, delays increase sharply when the user’s computation capability is low, reaching its maximum value. While the delay of the non-collaborative scheme gradually decreases, it remains higher than that of TD3, DDPG, and DQN, indicating that the lack of a collaboration mechanism limits its performance even as user computation resources improve. Therefore, the TD3 algorithm significantly optimizes user experience under varying local device computation capabilities and provides an effective solution for task optimization in low-computation- capability scenarios.

**Figure 11:**
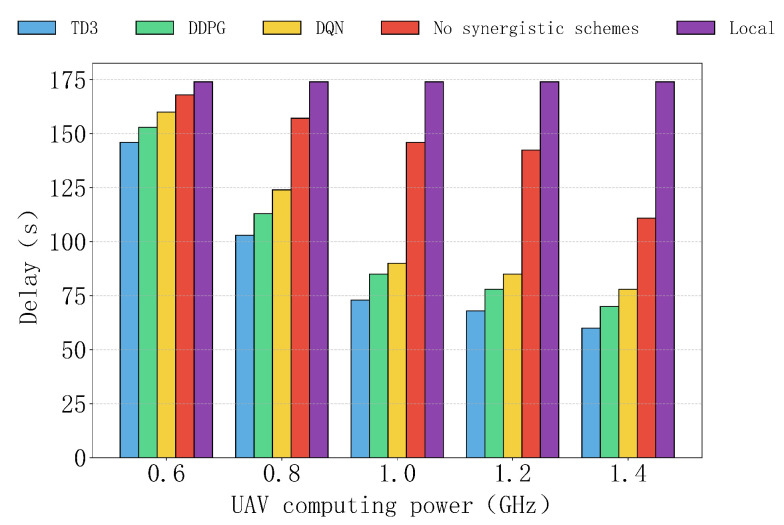
The impact of computing power of different UAVs on delay.

Figure 11 shows the delay performance of various algorithms, as well as the non-collaborative and local computation schemes, under different UAV computation capabilities. Overall, as UAV computation capabilities increase, the delay of the TD3 algorithm gradually decreases and outperforms all other schemes. In contrast, the delays of DDPG and DQN are relatively higher, but they still significantly outperform the non-collaborative and local computation schemes, especially in high computation capability scenarios. The local computation scheme, relying entirely on IoT devices, does not show a significant decrease in delay under different computation capability conditions. Although the delay of the non-collaborative scheme decreases with improved computation capability, due to the lack of a collaboration mechanism, its delay remains relatively high.

**Figure 12:**
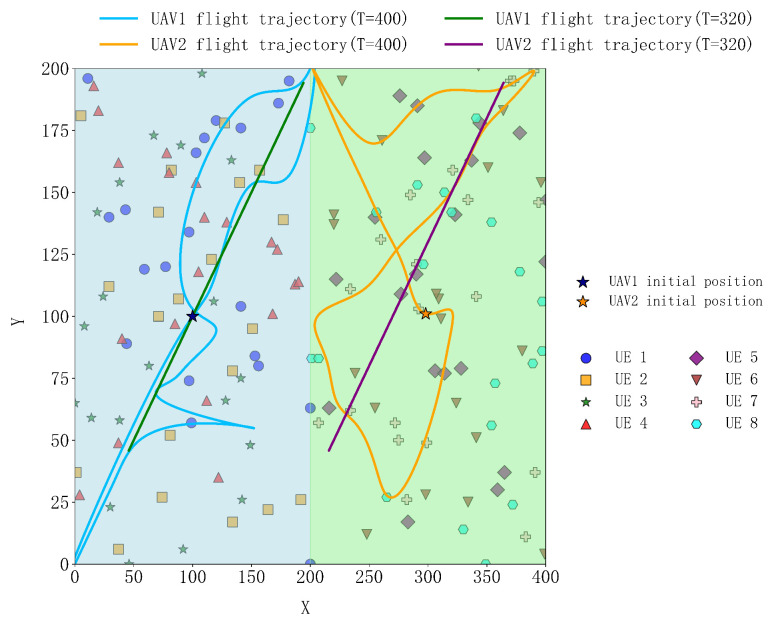
Two-dimensional flight trajectory of the UAV.

**Figure 13:**
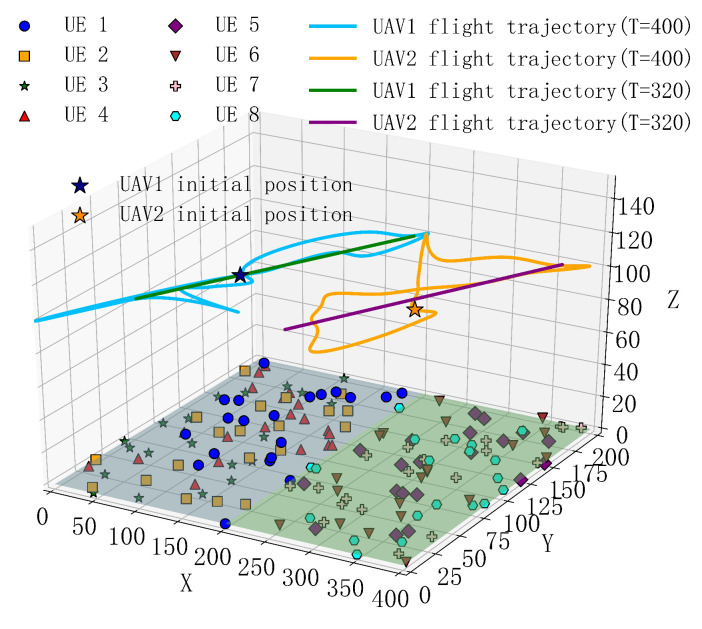
Three-dimensional flight trajectory of the UAV.

Figure 12 and Figure 13 show the 2D and 3D flight trajectory maps of the UAVs under different periods, where UE represents the ground IoT devices. From the figures, it can be observed that, in the case of a 400-s period, the UAV’s coverage area significantly expands due to the longer flight time. This allows the UAV to reach more ground IoT devices, providing offloading services for their computational tasks, reducing their load, and improving task processing efficiency. By flying to more distant terminal locations, the UAV can establish higher-quality communication links, thereby reducing task offloading time and optimizing the overall system performance.

In contrast, under the 320-s period, the UAV’s flight range is smaller, but it is still able to effectively cover the concentrated areas of users and provide efficient task offloading services. Despite the smaller flight range, the UAV trajectory planning for this period is more precise, allowing task offloading and processing to be completed in a shorter time, thus reducing communication and computation delays. In this case, by concentrating coverage on a local area, the UAV can optimize task allocation, avoid system overload, and reduce unnecessary communication overhead. Additionally, to make these trajectories more aligned with actual flight paths, the time slot data collected was smoothed. Through this smoothing process, the trajectory becomes smoother and more coherent, reducing jumps between sampling points, thereby enhancing the authenticity and visual effects of the trajectory. This processing not only improves the accuracy of the trajectory but also makes the simulation results closer to actual flight conditions.

Overall, the proposed scheme in this paper has significant advantages. The long-period coverage is wide and can provide computational offloading services for more users, reducing overall delay. Although the short-period coverage is smaller, it can serve more users distributed at greater distances in a shorter time, further optimizing system performance, reducing delays, and enhancing the user experience. This multi-period scheduling strategy enables the UAV to flexibly respond to different scenarios during task offloading, maximizing resource utilization efficiency.

To further evaluate the advantages of the TD3 algorithm in task offloading and trajectory optimization, this paper compares its average delay performance with that of the DDPG algorithm across various scenarios. As shown in Table 3, the TD3 algorithm consistently achieves significantly lower latency than the DDPG algorithm in all scenarios. Among them, the delay reduction in the task volume scenario is the largest, reaching 20.2%, the delay reduction in the flight speed and IoT computing power scenarios is 17.0% and 15.0%, respectively, and the delay reduction in the other scenarios is also more than 9.8%. The results show that the TD3 algorithm has significant optimization ability in the multi-user collaborative computing environment, which can effectively reduce the system delay and improve the real-time performance and stability of the system under high load and dynamic conditions.

Therefore, the task offloading and trajectory optimization method based on NOMA technology and multi-UAV collaboration proposed in this paper is superior to other existing schemes in reducing delay and improving mission offloading efficiency. By combining NOMA technology and TD3 algorithm, the unloading decision and flight trajectory of the UAV are optimized, which significantly improves the communication and computing capabilities of the UAV in a complex multi-user environment, balances the resource competition between multiple users, and effectively reduces the transmission queuing problem during task offloading.

## 5. Discussion 

This section discusses the contributions of each key component of the proposed framework and its practical implications for real-world deployment.

From the overall framework design and the comparative results, the roles of each component can be indirectly inferred. Specifically, the integration of NOMA directly enhances spectrum efficiency and multi-user access capability, thereby reducing queuing delays and supporting a larger number of IoT devices. The multi-UAV collaboration strengthens load balancing and service coverage, mitigating the limitations of a single node’s computing capacity. The TD3-based joint optimization further enables the system to dynamically adapt task offloading and trajectory planning according to real-time network states, achieving lower system latency compared to static or heuristic strategies.

In practical deployment, the proposed multi-UAV collaborative MEC framework integrated with NOMA could be applied in scenarios such as post-disaster rescue, large-scale outdoor events, and remote area connectivity, where rapid network setup and flexible computing support are required. In these environments, the UAV swarm’s ability to dynamically adjust its trajectories and balance computing tasks through cooperative offloading helps mitigate coverage holes and computing bottlenecks. However, practical challenges such as energy constraints, UAV flight safety regulations, and unpredictable weather conditions must be considered. To address these issues, the proposed joint optimization algorithm can be further extended by incorporating energy-aware trajectory planning and adaptive task scheduling strategies. This ensures that the system remains robust and responsive, even under varying real-world conditions.

Overall, these insights highlight not only the effectiveness of the proposed scheme but also the practical considerations and future improvements needed to bridge the gap between theoretical optimization and real-world UAV-assisted MEC applications.

## 6. Conclusions 

To address joint task offloading and trajectory optimization problem in UAV-assisted edge computing systems, in this paper, we propose a UAV-assisted edge computing system model that integrates NOMA technology and multi-UAV collaborative computing. To optimize system delay, a task offloading and trajectory optimization method based on the TD3 algorithm is designed. By establishing an information exchange mechanism between UAVs and IoT devices, our scheme alleviates UAV computational resource bottlenecks and the transmission queuing problem caused by multiple IoT devices offloading tasks simultaneously, thereby minimizing system delay. By transforming the non-convex optimization problem into an MDP decision-making problem, the offloading decisions and flight trajectories of UAVs are jointly optimized, effectively reducing computation delay in multi-IoT scenarios. Simulation results show that, compared to traditional approaches, our proposed scheme reduces delay by 20.2%, 9.8%, 17.0%, 12.7%, 15.0%, and 11.6% in scenarios with varying task volumes, the number of IoT devices, UAV flight speed, flight time, IoT device computing capabilities, and UAV computing capacities, respectively. In future work, we plan to extend the proposed framework to scenarios involving multi-task and heterogeneous UAV clusters. As network scale and task complexity continue to grow, a homogeneous UAV fleet may not be sufficient to handle diverse computing and sensing demands. To address this, we will explore the design of heterogeneous UAV systems that combine heavy-computation UAVs with lightweight reconnaissance drones, forming a flexible aerial cluster capable of efficiently coping with increasing ground device density and task loads.

## Figures and Tables

**Table 1:** Main symbols and their physical meanings.

Symbol	Physical Significance	Symbol	Physical Significance
*R*	Sub-region collection	$T_{k}^{Tr}\left(i\right)$	Near-end transmission delay
$K_{r}$	A collection of IoT devices within the region *r*	$T_{k}^{U}\left(i\right)$	Proximal computing delay
*j*	UAVs in region $J_{r}\in r$: edge UAVs	$E_{k}^{U}\left(i\right)$	The energy consumption of the UAV is calculated proximally
*o*	UAVs in region $J_{o}\in r$: cloud UAVs	$T_{k}^{O}$	The total delay of the proximal computation
$d_{kj}\left(i\right)$	Time-varying Euclidean distance from UAV *j* to IoT device *k*	$T_{k}^{Tr^{\prime }}\left(i\right)$	Cloud transmission delay
$d_{jo}\left(i\right)$	Time-varying Euclidean distance between UAV *j* and UAV *o*	$T_{k}^{U^{\prime }}\left(i\right)$	Cloud computing delay
$h_{k,j}\left(i\right)$	Channel gain from UAV *j* to IoT device *k*	$E_{k}^{U^{\prime }}\left(i\right)$	Calculate the energy consumption of UAVs remotely
$R_{k,j}\left(i\right)$	The transmission rate from UAV *j* to IoT device *k*	$T_{k}^{O^{\prime }}\left(i\right)$	The total delay of the remote computing
$R_{j,o}\left(i\right)$	The transmission rate of UAV *j* and UAV *o*	$T_{k}\left(i\right)$	The delay consumed by slot *i*
$T_{k}^{L}$	The local computing delay of IoT device *k*	–	–

**Table 2:** Main simulation parameters.

Parameter Name	Symbol	Valid Value	Parameter Name	Symbol	Valid Value
Number of IoT devices	*K*	4	Channel gain (UAV-UAV)	$\beta_{0}^{J}$	−50 dB
Number of drones	*J*	2	UAV-IoT bandwidth	$W_{k}^{j}$	1 MHz
Communication cycle	*T*	400 s	Inter-UAV bandwidth	$W_{j}^{o}$	1 MHz
Time slots	*I*	40 s	IoT-UAV transmission power	$P_{k}$	0.1 W
Time slot length	$t_{i}$	10 s	UAV-UAV transmission power	$P_{j}$	0.1 W
Flight area of UAV 1	$\left(x_{max},y_{max}\right)$	(200, 200) m	Gaussian noise power	$\sigma_{k}^{2}$	−100 dB
Flight area of UAV 2	$\left(x_{max},y_{max}\right)$	(400, 200) m	Battery capacity of UAV	$E_{UAV}$	500 kJ
Flight time	$t_{fly}$	20 s	IoT CPU	$f_{IoTD}$	0.6 GHz
Maximum flight speed	$v_{max}$	20 m/s	Computing power of UAV	$f_{uav}$	1.2 GHz
UAV quality	*M*	9.65 kg	Total task count	*D*	80 MBits
Channel gain (UAV-IoT)	*h*	−50 dB	-	-	-

**Table 3:** Comparison of the average delay performance.

Performance	Average Delay		Difference	Delay Reduction Rate
	TD3	DDPG		
Amount of tasks (MBits)	48.4 s	58.2 s	9.8 s	20.2%
Number of devices	90.6 s	99.5 s	8.9 s	9.8%
Flight speed (m/s)	64.8 s	75.8 s	11 s	17.0%
Flight time (s)	66.4 s	74.8 s	8.4 s	12.7%
IOT computing power (GHz)	67.8 s	78 s	10.2 s	15.0%
UAV computing power (GHz)	89.4 s	99.8 s	10.4 s	11.6%

## Data Availability

Data will be made available on request.
